# When people do not ‘Zol’: Reduced emergency centre attendance of patients with chronic obstructive pulmonary disease during coronavirus disease 2019 lockdown with the accompanying tobacco sales ban in South Africa

**DOI:** 10.4102/phcfm.v13i1.2750

**Published:** 2021-02-15

**Authors:** Piero Saieva, Louis S. Jenkins

**Affiliations:** 1 Department of Family and Emergency Medicine, George Regional Hospital, Western Cape Department of Health, George, South Africa; 2 Department of Family and Emergency Medicine, Faculty of Medicine and Health Sciences, Stellenbosch University, Cape Town, South Africa; 3 Directorate of Primary Health Care, Faculty of Medicine and Health Sciences, University of Cape Town, Cape Town, South Africa

**Keywords:** smoking, restrictions, COVID-19, COPD, emergency reductions

## Abstract

The coronavirus disease 2019 (COVID-19) pandemic has spread throughout the world, with devastating effects of the virus as well as the repercussions of the resulting ‘lockdowns’. South Africa went into a national lockdown in March 2020 to mitigate the impact of the virus. This included a ban on the sales of tobacco and electronic cigarette products. The ban has been a highly contentious issue in South Africa, discussed worldwide, which has drawn many criticisms. The prevalence rate of smoking in South Africa was around 21.5%, with the Western Cape province having a prevalence rate of 39%. We compared the number of chronic obstructive pulmonary disease (COPD) presentations at a large regional referral hospital in the Western Cape province from January to August 2019 with the same period in 2020. Electronic emergency centre data showed a reduction of 69.28% in COPD presentations. To control for some confounders for the same period, we also reviewed patients presenting with urinary tract infections, which showed only a 30.60% reduction. This notable reduction in COPD presentations reduced service pressure of emergency centre and most likely benefitted patients’ health. Further research and policies are needed to ensure ongoing reduction in the prevalence of smoking.

## Background

Smoking is a common practice across Africa, with the estimated prevalence rate of 14.35% by the World Health Organization (WHO).^1^ It was previously suggested that ‘Africa presents the greatest threat in terms of future smoking growth’.^2^ Cigarette smoking is known to be a major risk factor for developing and causing frequent exacerbations of chronic obstructive pulmonary disease (COPD).^3^ Chronic obstructive pulmonary disease has a prevalence rate of around 13.4% in Africa, with estimates of around 20% for South Africa (SA).^4,5^ Chronic obstructive pulmonary disease exacerbations have been linked to many triggers, such as change in season, air pollution, fires and smoking.

The coronavirus disease 2019 (COVID-19) pandemic was first reported in March 2020 in SA. During the same month, President Ramaphosa declared a national lockdown that restricted all services, except those that were deemed to be ‘essential services’.^6^ Alcohol and cigarettes were classified as ‘non-essential’, which led to a ban on legal tobacco sales.^6^ Cigarette smoking is a common practice in SA, with an estimated national prevalence rate of 21.5% and a prevalence rate of 39% in the Western Cape Province.^7^ The role of smoking in altering the risk of developing COVID-19 remains unclear, although it appears to increase the disease severity.^8^ The reasons for the tobacco sales ban were multifactorial, according to Dlamini-Zuma, the South African Minister of Cooperative Governance and Traditional Affairs. She argued that smoking not only increases the severity of COVID-19 pneumonia but also increases the strain on the healthcare system, with more beds needed for COVID-19 patients as well as the usual emergencies.^9^ The ban was met with mixed reviews because of economic and personal factors, with one of the criticisms being the estimated daily loss of R31 million in excise tax in SA.^10^

## Methods

### Setting

George Regional Hospital is a 272-bed regional referral hospital in the Garden Route district of the Western Cape Province in SA. The emergency centre (EC) manages 4000 patients monthly, and with the surrounding primary healthcare clinics, cares for 84% of the local community of 210 000 people.

### Sampling

All patients attending the EC are entered into an online electronic patient information system called ‘Hospital and Emergency centre tracking information system (HECTIS)’and receive a primary International Classification of Diseases ICD-10 diagnosis. Data on the total number of patients with COPD exacerbations attending from January to August in 2019 and for the same period in 2020 were extracted from HECTIS and analysed. We used the number of patients presenting with urinary tract infections (UTIs) during the same periods to control for possible confounders. January and February were included to show regular pre-lockdown figures for both the periods.

### Analysis

Simple statistics, including frequencies and percentages, were calculated and entered in an Excel^R^ spreadsheet.

### Ethical considerations

This article followed all ethical standards for a research without direct contact with human or animal subjects.

## Results

**FIGURE 1:**
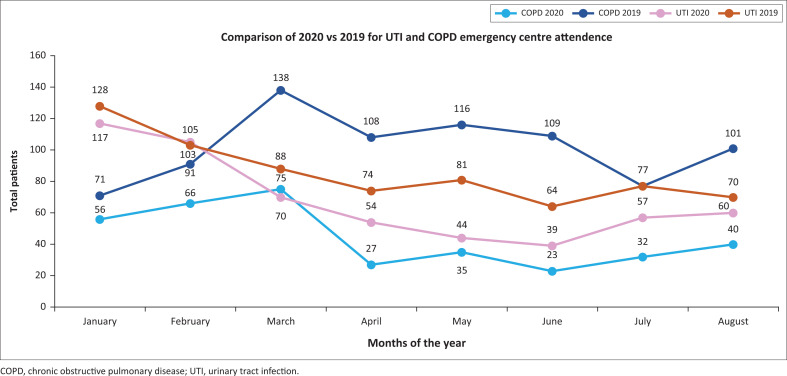
Comparison of patients presenting with chronic obstructive pulmonary disease and urinary tract infection at George Regional Hospital emergency centre.

Compared with 2019, there was a significant drop in EC attendance of COPD patients during the same period in 2020. In April 2020, there was a 75% (27 vs. 108) reduction compared with April 2019, in May 2020 a 70% (35 vs. 166) reduction, in June 79% (23 vs. 109), in July 58% (32 vs. 77) and in August there was a 61% (40 vs. 101) reduction. The combined reduction in COPD numbers during the year 2020 was 69.28% (see Figure 1).

With the national lockdown and the restrictions on movement, as well as the fear of accessing the hospital, we expected a decrease in patient numbers, as the hospital is the referral centre for severe COVID-19 infections in two districts. This was reflected in the HECTIS data that showed a reduction of patients presenting with UTIs, although not to the same extent as COPD numbers. In April 2020, there was a 27% (54 vs. 74) reduction in UTI cases, in May 46% (44 vs. 81), in June 39% (39 vs. 64), in July 26% (57 vs. 77) and in August 14% (60 vs. 70) reduction. The combined reduction in UTI numbers was 30.60% (see Figure 1).

## Discussion

The main finding was a 69.28% reduction in patients presenting to the EC with COPD exacerbations, compared with that during the previous year for the same period. For the same period, there was a 30.60%reduction in patient numbers presenting to the EC for UTIs. Whilst both patient numbers with COPD and UTIs decreased during the lockdown period with the accompanying tobacco sales ban, it is noteworthy that COPD attendance was markedly more reduced. Whilst acknowledging various limitations because of possible confounders, such as the obligatory wearing of masks, curfew and other factors, one cannot ignore these observations.

Although SA was one of a few countries to introduce a tobacco sales ban during the COVID-19 lockdown (India and Botswana did the same), many countries have instituted measures to restrict smoking habits, with suggested concomitant reductions in diseases like COPD.^11^ Ireland instituted a national workplace smoking ban in 2004, with a 15% decrease in overall pulmonary admissions.^12^ A Canadian study in 2010, declaring public and work spaces smoke free, showed a 33% decrease in admissions of patients complaining with respiratory conditions (such as COPD, asthma, pneumonia and bronchitis).^13^ Whilst there is limited data from African studies, the findings from this small study correlate with international research into how tobacco sales restrictions have reduced COPD exacerbations and EC attendances.

A University of Cape Town survey found that during lockdown 90% of previous smokers bought cigarettes.^14^ These cigarettes were bought at extremely elevated prices, with an average 20 pack of cigarettes costing R144, whereas prior to the lockdown 20 packs of cigarettes costed between R25 and R45, on average. Single cigarettes were reported to cost R5.69, on average. There was a rise in unemployment because of the lockdown, with many households losing their primary source of income. It, therefore, seems likely that this unusual situation would have forced smokers to reduce the amount they are smoking. The survey showed that of the 12 118 participants, 42% had attempted to quit smoking. Whilst only 39% had succeeded, which is not unexpected, yet it is a step in the right direction.^14^

## Conclusion

Whilst the tobacco sales ban has had detrimental effects on economy, one of the benefits has been a reduction in EC attendance of patients with COPD exacerbations, compared with that during the same period from the previous year. Whilst it is possible that the lockdown per se contributed to reduced EC attendance and reduced access to tobacco outlets or social gatherings, the net effect has been reduced pressure on the healthcare system because of a decreased number of patients attending the EC and needing admissions. We advocate for further research and policy into what confounders possibly played a role and which restrictions can be instituted to decrease the prevalence of smoking post lockdown, particularly in Africa.
